# A Network Comparison of Motives behind Online Sexual Activities and Problematic Pornography Use during the COVID-19 Outbreak and the Post-Pandemic Period

**DOI:** 10.3390/ijerph19105870

**Published:** 2022-05-12

**Authors:** Xiaoliu Jiang, Yingfei Lu, Youjuan Hong, Ying Zhang, Lijun Chen

**Affiliations:** 1 School of Humanities and Social Sciences, Fuzhou University, Fuzhou 350108, China; psyjxl@126.com (X.J.); sprifusoton@163.com (Y.L.); 2 School of Nursing, Fujian Medical University, Fuzhou 350108, China; yjhong_zp@126.com; 3 Department of Physical Education, Fuzhou University, Fuzhou 350108, China

**Keywords:** COVID-19, pornography use motivation (PUM), online sexual activities (OSAs), network analysis, community detection

## Abstract

Many researchers have considered whether online sexual activities (OSAs) increased over the course of the COVID-19 pandemic and whether these have led to an increase in problematic pornography use (PPU). This study investigated the impact of COVID-19 on PPU through pornography use motivations (PUMs) and OSAs to develop a better understanding of the mechanism and changes affecting PPU. Two groups of Chinese adults were recruited during the initial months of the pandemic (April 2020, *n*_1_ = 496) and the post-pandemic period (October 2021, *n*_2_ = 504). A network analysis was conducted to compare the structures of PPU symptoms among the two groups. The results showed that PUMs and OSAs were stronger predictors of PPU during the pandemic than post-pandemic (*R*^2^_pandemic_ = 57.6% vs. *R*^2^_post-pandemic_ = 28.7%). The motives of fantasy, sexual pleasure, stress reduction, and self-exploration were the prominent motivations during these two periods, but we found distinct PPU-related communities. PPU, sexual pleasure, and viewing sexually explicit materials (a type of OSAs) constituted a community during the pandemic but not in the post-pandemic’s network. The present study indicated that the pandemic may not have been the only factor impacting the higher rate of PPU. Instead, the higher frequency of OSAs during the pandemic may have been a strategy to cope with stress and to safely satisfy sexual desire.

## 1. Introduction

The revolution in information and internet communication technology (e.g., emergence of portable devices such as smart phones and laptops) has made pornography more convenient and prevalent worldwide [1,2,3]. In addition, pornography use is related to many aspects of our daily life, such as intimate relationships [4], sexual satisfaction, sexual problems [5], sexual aggression [6], and body image [7]. For instance, users’ sexual body image, penis-size dissatisfaction, and decisions concerning genital cosmetic surgery have been associated with pornography use [8,9]. A small percentage of people (5–14%) have been identified as having problematic pornography use (PPU) [10,11]. PPU has been characterized as persistent, repetitive use of pornography that results in the impairment in one’s life and the inability to reduce or cease PPU [7,9]; it is considered as a subcategory of compulsive sexual behavior disorder (CSBD) [12,13,14,15,16,17,18,19,20].

During the COVID-19 pandemic, online pornography consumption significantly increased [21]. Correspondingly, researchers discussed that COVID-19-related pornography-use patterns may have exposed many to a higher risk of developing or worsening PPU [21,22]. Data, though insufficient, supported that problematic use had increased along with the increase in pornography use during lockdowns [23], which suggested that the spike in pornography use may have been a coping strategy during the global pandemic. However, the empirical evidence has been limited. Furthermore, previous studies have been primarily conducted in western, educated, industrialized countries [23,24], it would be valuable to explore the pornography use in the eastern, conservative cultural countries such as China. Accordingly, comparing the characteristics of PPU over the initial months of the pandemic and during the post-pandemic era may contribute to better understanding the patterns of problematic and non-problematic engagement.

Furthermore, pornography use motivation (PUM) was a crucial variable in understanding PPU, especially during the pandemic. Motivation refers to the psychological process that promotes the behavior’s purpose and direction [25] and determines the likelihood and the intensity of a behavior [26]. Grubbs et al. mentioned that pornography use may be motivated by factors such as pleasure seeking and boredom reduction in the context of the pandemic [23]. However, sexual motivation is complex, with affective, motivational, and cognitive factors affecting the determination of engagement in sexual activity [27,28], especially as the COVID-19 pandemic and subsequent lockdowns impacted interpersonal and social behaviors as well as significant, unexpected changes in sexual habits [29,30,31,32]. Therefore, studying the characteristics of pornography use motivations may provide insights into pornography use within the context of the COVID-19 pandemic.

Although individual, solitary pornography use is the most common [33], other forms of online sexual activities such as those focused on mutual arousal with a partner [3,34,35], engaging in sex chats, and searching for sexual partners have also increased due to the use of social interaction via online media to fill the void of in-person activities during the pandemic [31,36]. Previous research found an association between craving pornography and the frequency of cybersex as watching pornography has often been identified as a gateway to cybersex, and vice versa [37]. More importantly, research has shown that both partnered and solitary activities could be problematic [38,39]. Considering that researchers have been encouraged to assess pornography use in a more comprehensive manner [40,41], both solitary and partnered OSAs were considered in the current study. 

### 1.1. Motives to Engage in OSAs 

Although the importance of exploring the motives behind using the Internet for sexual purposes has been well recognized [42,43,44,45,46,47,48], the prominent motivations for pornography use during the COVID-19 pandemic are not yet fully understood. Since the COVID-19 pandemic can undoubtedly be considered a major stressor for the global community [31,49,50], engaging in OSAs that avoided interpersonal contact were part of the safe recommendations for meeting sexual needs and coping with the ongoing stress without increasing the risk of contagion [28,51]. Moreover, researchers confirmed that people expand their sexual interests by engaging in OSAs during the pandemic [31]. It was plausible, therefore, that coping (to reduce stress or negative emotions) and sexual pleasure (satisfying sexual desire and sexual arousal) could have been the predominant motivations for OSAs during this period. In addition to the impact of sexual drives [52], however, identifying the salient characteristic of sexual motivations may further define the pornography use patterns during the COVID-19 pandemic.

### 1.2. Motives behind OSAs and Problematic Use

The motivations unpinning addictive behaviors have been intricately linked to the development of both behavioral and substance addictions [53,54,55]. Griffiths stated that the “motivations of people participating OSAs may further increase our understanding of Internet sex addiction” [56], and there is a growing body of research investigating the motives that fuel problematic and nonproblematic engagement in OSAs [38,48,57,58]. Several studies that have focused on this issue found that participants with higher levels of problematic use tend to have stress reduction and mood regulation motivators for their activities more frequently than participants with less problematic involvement in OSAs [38,46,48,58]. Previous studies found that participants with more problematic OSAs were more likely to be motivated by sexual pleasure [39,48]. Since both of these motivations have been associated with problematic use, we investigated whether the main motivation associated with PPU had changed at different time periods during the pandemic. In addition, although previous studies found that solitary and partnered OSAs frequently coexist and both can be associated with PPU [38,39], it was still unknown whether there were different motivations behind the varied types of OSAs. Conducting a study that clarified the complexity of these associations between different motives and types of OSAs could thus provide more insight into problematic use. 

### 1.3. Network Approach and Community Detection

Psychopathological states exist as dynamic, complex systems involving interactive components [59]. In contrast to some latent models, the network analysis method can mathematically analyze and visually display the relationships among complex variables [60]. More precisely, it can examine the interplay between different constructs [61] and provide a powerful visual depiction of complex associations that can reveal patterns and structures and efficiently identify variables at central positions in the network [62,63,64]. Network theories and methodologies have been successfully applied in studies concerning hypersexuality [63] and PPU [65,66]. In the current study, we used the network approach to assess PPU-related network topology in order to identify the most prominent motive, and node communities in symptom domains during the pandemic and post-pandemic periods and to provide a novel perspective from which to examine whether pornography use may be a problematic coping strategy during emergency situations.

### 1.4. The Current Study

By examining the patterns of PUMs and OSAs during and after the COVID-19 pandemic, this study considered the influence of COVID-19 on these factors to better understand the characteristics of problematic engagement. We used data from two groups, separately recruited, during the initial months of the pandemic and the post-pandemic era to address the following objectives: (1) to compare the network topology and predictability of two PPU-related networks, (2) to identify the most salient characteristic of the motivations in the two networks, and (3) to examine the clusters of PUMs and OSAs and whether they were associated with problematic use during the two different time periods.

## 2. Materials and Methods

### 2.1. Participants and Procedure

Pandemic group: Participants were asked to complete an anonymous Internet-based survey through a popular Chinese survey website Wenjuanxing in April 2020, for which each participant received a compensation of CNY 15. Eligible participants had to (a) be 18 years old or older; (b) report having engaged in OSAs at least once during the past 3 months; and (c) reside in China. A total of 496 adult respondents (females = 240, 48.39%) from 29 of the 34 provinces in China were included in the final data set. A total of 95 respondents were excluded due to disqualifying information (e.g., incomplete or duplicate data, younger than 18, did not engage OSAs in the past 3 months).

Post-pandemic group: Using the same survey website with the same inclusion and exclusion criteria, a total of 504 adult respondents (females = 251, 49.80%) who had engaged in OSAs at least once during the past 3 months were recruited in October 2021. In August, September, and October 2021, 31 provinces (including autonomous regions and municipalities) in Mainland China (China’s population is approximately 1.4 billion) reported monthly COVID-19 confirmed cases of 1081, 1264, and 1893, respectively (data retrieved from the National Health Commission of the People’s Republic of China), and cross-provincial travel had been reinstated. Participants were recruited from 28 of the 34 provinces in China. Table 1 summarizes the sociodemographic characteristics of the two groups. The study was approved by the local ethics committee of Fuzhou University.

### 2.2. Instruments

The pornography use motivations scale (PUMs) was described in [46]. The PUMs is a 24-item self-report scale that assesses eight dimensions of PUM factors, each with three items: sexual pleasure, sexual curiosity, fantasy, boredom avoidance, lack of sexual satisfaction, emotional distraction or suppression, stress reduction, and self-exploration. Respondents indicated their answers on a 7-point scale from 1 (never) to 7 (all the time). The PUMs showed good reliability in both groups (α = 0.97 in the pandemic group, and α = 0.95 in the post-pandemic group).

The online sexual activity questionnaire was described in [3]. Thirteen items were used to measure the use of the Internet in four dimensions: viewing sexually explicit materials (SEM), seeking sexual partners, cybersex, and flirting and sexual relationship maintenance. Assessed items were rated on a 9-point scale that ranged from 1 (never/0 time) to 9 (at least once a day/20 or more times). Higher scores were indicative of more frequent engagement in OSAs. Cronbach’s alpha of the entire scale was 0.91 in the pandemic group and 0.82 in the post-pandemic group.

The brief pornography screening (BPS) is a short screening tool that measures self-control and use of pornography to identify individuals at risk of PPU [67]. This five-item scale uses a 3-point rating scale, and a cut-off score of 4 was suggested to detect impaired control (range = 0–10). Higher scores indicate higher levels of impaired control regarding pornography use. Cronbach’s alpha of the entire scale was 0.88 in the pandemic group and 0.90 in the post-pandemic group.

### 2.3. Analysis

Statistical analyses were carried out in SPSS 22.0 (IBM, Armonk, NY, USA) and R version 4.1.1 (R Foundation for Statistical Computing, Vienna, Austria), using mgm, bootnet, qgraph, network comparison test, and igraph packages to compute the networks. We estimated and analyzed the networks in three sections. All analyses were carried out separately for each group.

To investigate how the twelve predictors (i.e., the four dimensions of OSAs and eight dimensions of PUMs) were associated with PPU (measured by BPS), all thirteen variables were included in a mixed graphical model (MGM) using the mgm package [68]. Least absolute shrinkage and selection operator (LASSO) regression was adopted for adjustment to reduce the appearance of false connections. To choose the most appropriate network, we used the extended Bayesian information criterion (EBIC) and selected γ = 0.5 as the hyperparameter value to ensure a more conservative network estimation [69,70]. We visualized the estimated network models using the qgraph package [71] and also calculated the node predictability for statistical comparison (was interpreted akin to *R*^2^) to provide the variance of each node that could be explained by all other nodes in the network [68,72]. 

Next, to identify the most prominent motivations during these two periods, we calculated the three most popular indices of node centrality [73,74,75]. Case-dropping subset bootstrap was used to test the accuracy of node centrality, and the correlation stability (*CS*) coefficient was considered as the selection criteria (≥0.5 for excellent fit) [73]. The network comparison test (NCT) was used to calculate *p*-values per node from the permutation test concerning differences in centralities [76].

Lastly, to explore differences in the structure of connectivity among PUMs, OSAs, and PPU across groups from an additional perspective, we ran a community detection algorithm. A community is a cluster of nodes (e.g., factors or variables) that have more strongly connected subnetworks in the network and can identify a group of nodes that are primarily affected when a node in the respective subnetwork changes states. We used the spinglass algorithm derived from principles of statistical mechanics [77] with the igraph package [78]. The following parameters were used: spins = 25, γ = 1, start temperature = 1, stop temperature = 0.01, cooling factor = 0.99.

## 3. Results

### 3.1. Characteristics of the Pandemic and Post-Pandemic Groups 

Regarding sociodemographic characteristics, the results of the chi-squared tests indicated that these two groups did not significantly differ from each other, except for relationship status (see Table 1), which suggested that the two groups were demographically comparable. The descriptive statistics for scores concerning the PUMs, OSAs, and BPSs are presented in Table 2. The proportion of participants above the cutoff score of the BPS (≥4) was 61.69 percent in the pandemic group and 40.28 percent in the post-pandemic group. Overall, the public had significantly stronger sexual motivations, engaged in OSAs more frequently, and were at higher risk of PPU during the initial months of the pandemic than in the post-pandemic period (*p*s < 0.001), with the exception of one OSA dimension, viewing SEM. On all variables, males scored significantly higher than females during the pandemic and post-pandemic periods (*p*s < 0.05). Moreover, as the results of chi-squared show in Table 2, there was a higher proportion at risk for PPU during the COVID-19 pandemic than during the post-pandemic, regardless of gender.

### 3.2. Network Analysis

#### 3.2.1. Network Structure and Predictability

**Figure 1:**
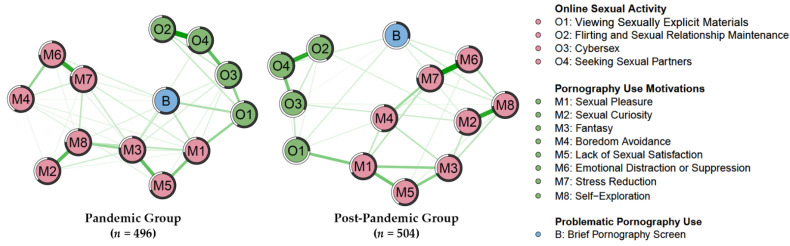
Network topology for PUM, OSA, and BPS by group. Green color edges represent positive, and red color edges represent negative associations, all lines in both networks are green. Thickness and saturation of edges indicate the strength of associations. The filled part of the pie chart around each node shows the predictability of each node, representing the variance of the nodes explained by other nodes in the networks.

**Figure A1:**
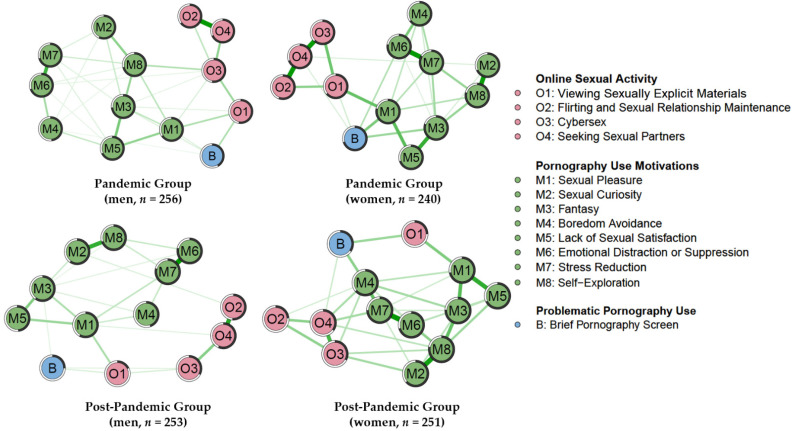
Network topology for PUM, OSA, and BPS by gender.

The MGM networks for the two groups are presented in Figure 1. Visual inspection suggested that BPS occupied the center of the pandemic group’s network with high predictability (*R*^2^ = 0.58); in contrast, it occupied the peripheral position in the post-pandemic group’s network (*R*^2^ = 0.29). In addition, the nodes in the pandemic group’s network had a higher average predictability than those in the post-pandemic group. The nodes explained, on average, 66.8% of the variance between them in the pandemic network and 54.2% of the variance in the post-pandemic network. Similar tendencies were observed in both male and female nets (Figure A1 and Table A1 in Appendix A contained additional details), indicating that OSA and motivation were more predictive of PPU during the pandemic than afterward. Predictability represents how strongly a focal node is influenced by its neighbors in an individually estimated network; however, it does not provide visual details as to which node is more important within a network and whether there is a difference in importance for a given node in jointly estimated networks. Fortunately, node centrality helped to identify the most prominent nodes during these two periods.

#### 3.2.2. Node Centrality

**Figure 2:**
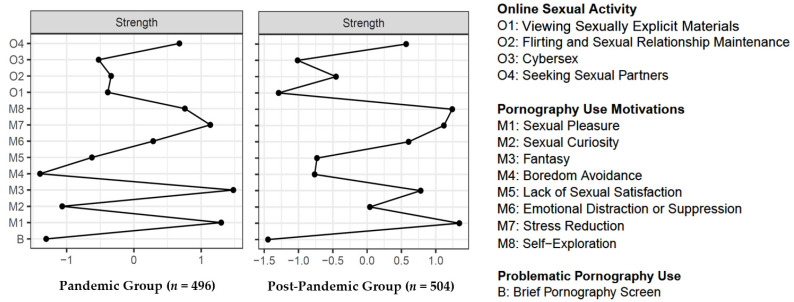
Node centrality plot of strength.

**Figure A2:**
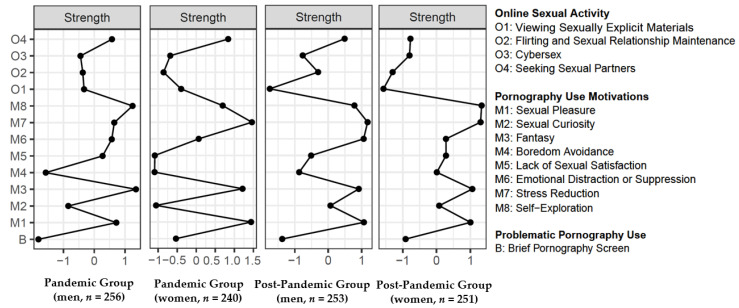
Node centrality plot of strength by gender.

Bootstrap stability analyses indicated that the closeness and betweenness (*CS* coefficients < 0.5) estimates were not as stable as strength (*CS* coefficients = 0.75). According to the recommended value of the stability coefficient [73], we limited the interpretation of centrality statistics to strength centrality (i.e., the absolute sum of edge weights attached to a node). The z-score value of the strength for each node in the network is shown in Figure 2. There was a high similarity in the pattern of node centrality for the pandemic and post-pandemic groups. Specifically, using the NCT, we found that nodes with the highest strength centrality estimates were sexual pleasure (M1, diff = −0.02, *p* = 0.859), stress reduction (M7, diff = 0.01, *p* = 0.956), fantasy (M3, diff = 0.13, *p* = 0.105), and self-exploration (M8, diff = −0.08, *p* = 0.300), in both networks. Nevertheless, NCT showed that there were significant differences on the point of O1 (viewing SEM, diff = 0.28, *p* = 0.004) and BPS (diff = 0.17, *p* = 0.005). BPS was significantly higher in the pandemic group than in the post-pandemic group, suggesting that the public was more likely to overuse pornography during an emergency, but could reduce their use once the emergency had passed. Although the frequency of viewing SEM during the pandemic period was not significantly higher than it was during the post-pandemic, the node centrality showed it was more prominent during the pandemic, which indicated the frequency of viewing SEM should not be overlooked while under stress. Furthermore, we conducted brief centrality estimates of the two groups in both genders. In all four networks (please see Figure A2 in Appendix A for more information), the nodes with the highest estimated strength centrality were still sexual pleasure (M1), stress reduction (M7), fantasy (M3), and self-exploration (M8). The motivation of emotional distraction or suppression appeared more prominent for males, both during the pandemic and post-pandemic, but it was marginally significantly different for females (diff = 0.15, *p* = 0.069, please see Figure A2 in Appendix A).

#### 3.2.3. Network Communities

**Figure 3:**
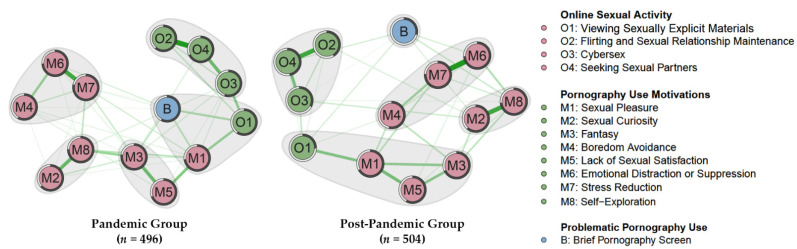
Community of nodes based on spinglass algorithm, visualized on the MGM networks by group.

Since centrality could not determine which types of motivations and activities were more strongly associated with BPS, the community detection method was applied. The results of the spinglass cluster detection algorithm are presented in Figure 3. The analysis pointed to five distinct communities (i.e., clusters) of nodes in both networks, and the composition of the communities was similar across groups. The four dimensions of OSAs were generally clustered into two clusters, which could be classified as either solitary arousal (O1) and partnered arousal (O2–O4) activities. The eight dimensions of PUMs could be roughly divided into three clusters, including pleasure-seeking motives (M1, M3, M5), information-seeking motives (M2, M8), and coping motives (M4, M6, M7). Most noteworthy was that one community comprised the BPS, sexual pleasure, and viewing SEM (O1) in the pandemic network. In contrast, BPS formed a cluster on itself in the post-pandemic network, and the community comprising sexual pleasure and viewing SEM were distant while coping motives and partnered-arousal activities were relatively closer to the solo BPS cluster. 

**Figure A3:**
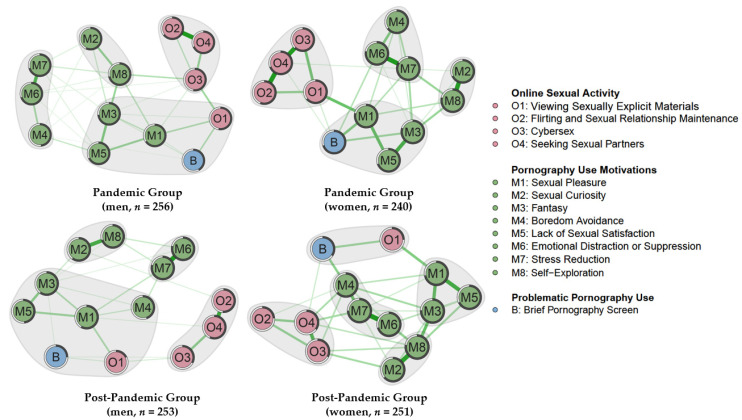
Community of nodes based on spinglass algorithm, visualized on the MGM networks by gender.

When we stratified the community detection by gender, pleasure-seeking (M1, M3, M5) and solitary-arousal (O1) were closer to BPS for males, and these five nodes comprised a community for both networks. Comparatively, for females during the pandemic, the BPS and pleasure-seeking clusters worked as a community; during the post-pandemic, the BPS and solitary-arousal (O1) clusters constituted a community, and the coping clusters (M4, M6, M7) were closer to the BPS (see Figure A3 in Appendix A).

## 4. Discussion

The objective of the current study was to observe, study, and understand the characteristics, motivations, and behaviors associated with the problematic use of pornography during and after the pandemic in order to provide insights for the evaluation of and preventative measures for PPU. This study found that (1) the public had significantly stronger sexual motivation, engaged in more frequent online sexual activities, and were more likely to be a higher risk of PPU during the pandemic than in the post-pandemic period; (2) centrality estimates indicated that the core nodes were fantasy, sexual pleasure, stress reduction, and self-exploration in the networks of both groups; and (3) there were distinct communities (clusters) of nodes associated with PPU: sexual pleasure, viewing SEM, and PPU formed one community in the pandemic network, whereas in the post-pandemic network, PPU formed a cluster on its own and was closer to the regulating (negative) emotion cluster but farther from the sexual pleasure cluster. 

Descriptive statistics and *t*-tests revealed that people had significantly stronger motivations, had OSAs more frequently, and had a higher risk for PPU during the initial months of the pandemic than in the post-pandemic era. Interestingly, the frequency of viewing SEM remained stable across groups, but the BPS scores dropped. This finding was attributed to viewing SEM being typical for many adults and adolescents, and its popularity rivals other modern technology-based activities (e.g., playing video games) [79]. This finding also indicated that as a quantitative indicator of PPU, the frequency of pornography use would not be a sufficient criterion for screening PPU [41]. There was also a difference in the network structures of these two groups. The PPU (measured by BPS) was positioned centrally with high predictability in the pandemic network, but not in the post-pandemic network. These were consistent with the results found by Grubbs et al., and their data showed that following the height of the first wave of the pandemic-related lockdowns, people reported higher pornography use than during other time periods [23]. However, compared with the available data from our previous studies, people engaged in OSAs more frequently at the peak of the pandemic and then returned to similar levels found prior to the COVID-19 pandemic [17,66,80,81]. China was the first country hit by the pandemic [82]. The sudden, large-scale infectious hazard, the strict isolation measures, and the unpredictable future placed increased pressure and severe psychological distress on the population of China during the initial months of the COVID-19 pandemic [49,83]. Health care providers suggested that sexual activity via digital platforms was the optimal choice during the pandemic (in addition to abstinence) [51,84], so it was expected that people would engage in more OSAs to meet their sexual needs and cope with the ongoing stress, even in conservative cultures. However, intense sexual desire and increased engagement in OSAs has been be related to the development of problematic use [38,80,85], especially in sexually conservative cultures where there has been a stronger association between the quantity and the severity of pornography use [41]. 

Node centrality analyses identified the motivations of fantasy, sexual pleasure, stress reduction, and self-exploration as the most central nodes in both networks. Fantasy is an intentional act of imagination that is usually solitary, typically resulting in daydreaming or masturbation [86], and it can serve the purpose of pleasure [87]. As expected, previous studies suggested that sexual pleasure and stress reduction were the main motives that drove people to engage in OSAs when confronted with the COVID-19 pandemic. Higher levels of psychological distress often result in a greater propensity to use pornography [88]. Given the evidence that many people experienced considerable economic hardship, acute stress, anxiety, and depression associated with the disruptive nature of the pandemic [83,89,90], using OSAs to cope with stress, unpleasant mood states, and boredom was reasonable [45,46,88]. Our finding that there was no difference in motivations during the pandemic and post-pandemic periods also suggested that the influence of the COVID-19 pandemic on mental health remained despite being nearly under control in China. As researchers predicted, individual and societal effects caused by COVID-19, such as economic distress and unemployment, would continue for several years after the pandemic [91,92]. For example, workers may experience increased stress, including exposure to the virus itself, changes in work arrangements and schedules, effects on work–life balance, lack of social support at work, and loss of income [93,94,95]. 

Self-exploration refers to a motivation to gain knowledge regarding one’s sexual desires and preferences [46,96]. Although information-seeking was not the most common motivation for pornography use [43], it appeared to be enhanced in the current study. A possible interpretation of this finding was the impact of culture on sexual motives and sexual education [97,98]. As individuals could use OSAs as a source to promote sexual health through greater sexual exploration, mutual disclosure, and instant access to sexual information and education [99,100,101,102], they were more likely to engage in them, particularly in Asian countries that hold conservative views concerning sexuality and lack quality sex education [103,104]. 

We also found specific patterns of motivations and activities associated with the PPU during the two periods. During the initial months of the pandemic, solitary OSAs were directly associated with PPU and driven by the motivation of sexual pleasure. Sexual pleasure-seeking motives were commonly reported as predominant reasons for pornography consumption [38,43,45,46,88]. Solitary arousal OSAs, which typically involve viewing pornography, have separated sexual pleasure from intimacy in relationships and allow people quickly to focus on pleasure-motivated goals such as sexual fantasies [35,38], which had been suggested as a safe approach during the pandemic [105]. Although high-frequency pornography use might not be a sufficient indicator of PPU [65,106,107], it could reflect the degree of PPU to some extent [80]. 

As the consumption of pornography for pleasure increased rapidly within a short period of time during the pandemic, individuals may have been aware of their excessive engagement and their risk of PPU. As the BPS [67] was a sensitive tool for assessing self-control and the use of pornography to identify individuals at risk of PPU, the specific pattern of the PPU-related subnetwork was captured. However, this pattern appeared to be more of a short-term adaptation during the COVID-19 pandemic. During the post-pandemic period when individuals gradually returned to normal life activities, PPU was more closely linked to the clusters of partnered OSAs and coping motives rather than pleasure-seeking motives. However, solitary OSAs were still associated with the pursuit of sexual pleasure. Coping motivations were particularly concerning, as pornography use in response to negative emotions has been associated with more problematic use (i.e., compulsive, dysregulated, or excessive use) of pornography in general [46,88]. In addition, agreeing with previous research findings regarding problematic engagement in OSAs [38,39], our results suggested that both partnered activities and solitary activities were associated with problematic use; however, the closeness of the association could depend on the heterogeneity of motives behind different types of OSAs. Therefore, the current study provided evidence as to the importance of considering different types of OSAs and motives in problematic expression. 

Considering that there have been gender differences in pornography-use variables, such as frequency of use, types of OSAs, use motivation, the proportion of PPU, a gender difference analysis was conducted in network structure, node centrality, and community detection. The results showed that similar structures were observed in both male and female nets, during outbreak and post-pandemic. The motivations of pornography use were stable for both genders; nevertheless, emotional distraction or suppression was more prominent for males, especially during the post-pandemic period. This finding was in line with previous research, where males were more likely to use pornography to reduce negative feelings [46]. Male pleasure-seeking motives and solitary arousal activities have been related more closely to PPU both during the pandemic and post-pandemic periods, suggesting that pleasure-seeking and solitary arousal activities were more likely to result in PPU. This finding was consistent with previous studies regarding sexual desire and craving pornography. In general, as males tend to have stronger sexual desire than females, and as increased sexual desire has been related to pornography use for pleasure-seeking motivation [108,109], males have shown stronger cravings for pornography [110]. Comparatively, for females, the variables closer to PPU changed at different pandemic stages. During the pandemic, similar to males, pleasure-seeking motives were closer to PPU. Existing data also supported the results as PPU was mostly predicted by sexual pleasure motives among female participants and by mood regulation motives among male participants [48]. During the post-pandemic period, solitary arousal and coping motives were closer to female PPU. This result was attributed to the impact of COVID-19 on the labor market: more females than males lost their jobs, more females than males were in essential jobs that exposed them to risk and psychological stress, and females experienced more work disruption than males due to expectations surrounding childcare and other responsibilities [111]. 

Some limitations should be considered when interpreting our findings. First, though the two groups from different time periods of the pandemic had similar demographic variables, they were cross-sectional groups that did not allow for causal inference. To fully explore the impact of COVID-19 on PPU, it is crucial to explore the progressive development of OSAs into PPU based on longitudinal studies. Second, the groups were self-selected via online surveys and the instances of bias (e.g., higher average educational attainment) in the group selection could have affected the results and limited the generalization of the results. Third, a short unidimensional measurement, the BPS [67], was used to assess PPU in the current study. The BPS focuses on measuring the loss control of pornography use, and it has been recognized as the gold standard for short screenings [81]. While the brevity of the BPS is a benefit, there may be gaps between the multidimensional and unidimensional measurements in screening PPU. Using a measure assessing a broader variety of PPU symptoms, such as the problematic pornography consumption scale [112], could provide more insights into PPU.

## 5. Conclusions

This study examined the motivations underpinning the OSAs associated with PPU, and these differed at different time periods during the pandemic. During the initial months of the pandemic, solitary OSAs motivated by sexual pleasure were a major risk factor for PPU, while different patterns of partnered OSAs motivated by coping needs were closely linked to PPU in the post-pandemic period. Future research is encouraged to further investigate the role of specific motives and OSA clusters in the development and maintenance of PPU.

## Figures and Tables

**Table 1:** Comparisons of the two groups on demographic characteristics.

Characteristic		Pandemic Group (*n* = 496)	Post-Pandemic Group (*n* = 504)	χ^2^ (*F*)	*p*
Gender ratio (men/women)		1.07	1.01	0.200	0.655
Age	Mean ± SD	29.46 ± 6.79	29.42 ± 6.29	0.075	0.940
	Range	18–52	18–51	-	-
Sexual orientation	Homosexual	0.60%	1.19%	0.962	0.618
	Heterosexual	98.39%	97.82%		
	Bisexual	1.01%	0.99%		
Relationship status	Single	10.08%	21.03%	22.770	<0.001
	Partnered	89.92%	78.97%		
Educational level	Primary school or below	0	0.40%	2.661	0.447
	Vocational school	1.21%	0.79%		
	Middle school	2.02%	1.59%		
	University or college	96.77%	97.22%		
Place of residence	Capital	55.65%	57.74%	2.438	0.487
	County town	40.12%	39.49%		
	Town	2.42%	1.98%		
	Village	1.81%	0.79%		

**Table 2:** Descriptive statistics and *t*-tests on measures of PUM, OSA, and BPS (Mean ± SD).

Variables		*t*/χ^2^^a^	Pandemic (*n* = 496)	Men (*n* = 256)	Women (*n* = 240)	*t*/χ^2^^b^	Post-Pandemic (*n* = 504)	Men (*n* = 253)	Women (*n* = 251)	*t*/χ^2^^c^
The average score of PUM (1–7)		7.745 ***	3.54 ± 1.20	3.80 ± 1.08	3.27 ± 1.26	4.98 ***	2.96 ± 1.18	3.21 ± 1.08	2.71 ± 1.22	4.78 ***
	M1 Sexual Pleasure	5.393 ***	3.84 ± 1.33	4.11 ± 1.19	3.55 ± 1.42	4.72 ***	3.37 ± 1.43	3.69 ± 1.27	3.05 ± 1.50	5.20 ***
	M2 Sexual Curiosity	8.507 ***	3.54 ± 1.33	3.73 ± 1.23	3.33 ± 1.41	3.40 ***	2.81 ± 1.41	3.03 ± 1.43	2.58 ± 1.35	3.67 ***
	M3 Fantasy	5.046 ***	3.53 ± 1.45	3.81 ± 1.38	3.25 ± 1.47	4.40 ***	3.07 ± 1.47	3.31 ± 1.42	2.83 ± 1.48	3.68 ***
	M4 Boredom Avoidance	3.263 ***	3.30 ± 1.30	3.54 ± 1.21	3.04 ± 1.35	4.32 ***	3.02 ± 1.44	3.33 ± 1.41	2.71 ± 1.41	4.96 ***
	M5 Lack of Sexual Satisfaction	4.094 ***	3.33 ± 1.36	3.57 ± 1.29	3.08 ± 1.40	4.10 ***	2.97 ± 1.47	3.24 ± 1.47	2.69 ± 1.42	4.27 ***
	M6 Emotional Distraction or Suppression	7.568 ***	3.42 ± 1.44	3.68 ± 1.35	3.16 ± 1.49	4.03 ***	2.74 ± 1.44	2.94 ± 1.39	2.54 ± 1.47	3.08 **
	M7 Stress Reduction	8.524 ***	3.58 ± 1.45	3.91 ± 1.33	3.23 ± 1.49	5.39 ***	2.81 ± 1.42	3.01 ± 1.36	2.61 ± 1.46	3.17 **
	M8 Self-Exploration	9.510 ***	3.76 ± 1.44	4.00 ± 1.35	3.51 ± 1.49	3.84 ***	2.90 ± 1.43	3.09 ± 1.41	2.71 ± 1.43	3.05 **
The average score of OSA (1–9)		8.347 ***	2.63 ± 1.21	2.92 ± 1.21	2.32 ± 1.13	5.64 ***	2.08 ± 0.82	2.29 ± 0.91	1.88 ± 0.66	5.72 ***
	O1 Viewing SEM ^d^	0.865	3.32 ± 1.49	3.81 ± 1.45	2.80 ± 1.35	8.04 ***	3.24 ± 1.45	3.66 ± 1.31	2.81 ± 1.47	6.89 ***
	O2 Flirting and Sexual Relationship Maintenance	7.153 ***	2.51 ± 1.61	2.68 ± 1.68	2.32 ± 1.53	2.48 *	1.86 ± 1.21	2.00 ± 1.42	1.72 ± 0.93	2.68 **
	O3 Cybersex	5.384 ***	2.04 ± 1.31	2.18 ± 1.41	1.89 ± 1.18	2.50 *	1.64 ± 1.00	1.78 ± 1.18	1.50 ± 0.76	3.20 ***
	O4 Seeking Sexual Partners	7.701 ***	2.21 ± 1.43	2.39 ± 1.54	2.00 ± 1.27	3.04 **	1.60 ± 1.05	1.69 ± 1.23	1.50 ± 0.81	2.07 *
The total score of BPS (0–10)		7.253 ***	4.53 ± 2.65	5.21 ± 2.52	3.81 ± 2.59	6.10 ***	3.30 ± 2.72	4.08 ± 2.69	2.51 ± 2.52	6.78 ***
The proportion that reached the cutoff score (≥4) of BPS		45.88 ***	61.69%	73.83%	48.75%	32.97 ***	40.28%	49.41%	31.08%	17.60 ***

* *p* < 0.05, ** *p* < 0.01, *** *p* < 0.001. ^a^ *t*-test or chi-square test was used to determine whether there was a difference in the two groups; ^b^ *t*-test or chi-square test was used to determine whether there was a gender difference in the pandemic group; ^c^ *t*-test or chi-square test was used to determine whether there was a gender difference in the post-pandemic group; and ^d^ SEM = sexually explicit materials.

## Data Availability

The data presented in this study can be requested from the authors.

## Appendix A

**Table A1:** Explained variances (*R*^2^) for variables included in the networks.

Variables	Pandemic Group (*n* = 496)	Men (*n* = 256)	Women (*n* = 240)	Post-Pandemic Group (*n* = 504)	Men (*n* = 253)	Women (*n* = 251)
M1 Sexual Pleasure	0.73	0.65	0.79	0.67	0.58	0.72
M2 Sexual Curiosity	0.63	0.57	0.68	0.61	0.60	0.63
M3 Fantasy	0.78	0.73	0.80	0.68	0.60	0.76
M4 Boredom Avoidance	0.58	0.48	0.65	0.53	0.45	0.63
M5 Lack of Sexual Satisfaction	0.69	0.68	0.70	0.58	0.49	0.68
M6 Emotional Distraction or Suppression	0.74	0.69	0.78	0.74	0.79	0.70
M7 Stress Reduction	0.78	0.70	0.84	0.76	0.79	0.75
M8 Self-Exploration	0.75	0.71	0.79	0.73	0.70	0.78
O1 Viewing SEM	0.59	0.53	0.64	0.26	0.18	0.25
O2 Flirting and Sexual Relationship Maintenance	0.61	0.64	0.56	0.41	0.51	0.24
O3 Cybersex	0.55	0.52	0.62	0.33	0.33	0.33
O4 Seeking Sexual Partners	0.68	0.68	0.69	0.47	0.56	0.35
BPS	0.58	0.42	0.70	0.29	0.25	0.35
Mean Explained Variance	0.67	0.62	0.71	0.54	0.52	0.55
